# Efficacy of levetiracetam for neonatal seizures in preterm infants

**DOI:** 10.1186/s12887-018-1103-1

**Published:** 2018-04-10

**Authors:** Ji Yoon Han, Chung Joon Moon, Young Ah Youn, In Kyung Sung, In Goo Lee

**Affiliations:** Department of Pediatrics, College of Medicine, Seoul St. Mary’s Hospital, The Catholic University of Korea, 222 Banpodaero, Seochogu, Seoul, 137-701 South Korea

**Keywords:** Levetiracetam, Neonatal seizures, Preterm infants

## Abstract

**Background:**

Neonatal seizures remain a significant clinical problem, and therapeutic options are still not diverse with limited efficacy. Levetiracetam (LEV) is a relatively new and wide spectrum anti-seizure medication with favorable pharmacokinetics and safety profile. In the recent decades, LEV has been increasingly used for the treatment of neonatal seizures. The aim of this study was to describe the experience of using LEV as the first line anti-seizure medication for preterm infants.

**Methods:**

A retrospective analysis of 37 preterm infants who were treated with LEV as the first-line anti-seizure medication was performed.

**Results:**

Mean gestational age of the 37 preterm infants was 31.5 ± 1.9 weeks (range, 26 to 36^+ 6^ weeks). Twenty-one infants (57%) were seizure-free while given LEV at the end of the first week, and no additional anti-seizure medication was required. Loading doses of LEV ranged from 40 to 60 mg/kg (mean 56 mg/kg) and the maintenance dose ranged from 20 to 30 mg/kg (mean 23 mg/kg). No adverse effect was observed.

**Conclusions:**

Levetiracetam can be a good and safe choice for treatment of neonatal seizures in preterm infants. Prospective double blind controlled studies are needed in the future.

## Background

Neonatal seizures are clinically defined as abnormal, stereotyped and paroxysmal dysfunctions in the central nervous system (CNS), occurring within the first 28 days after birth in full-tem infants or before 44 weeks of gestational age in preterm infants. It is the most common neurological event in neonates, indicating a variety of different pre-, peri-, or postnatal diseases of the CNS. Seizures occur more often during the neonatal period compare to other periods in the life span. They affect up to 1.5–3.5/1000 in full-term infants and 10–130/1000 in preterm infants [1], with a consistently identified trend a greater risk for seizures in preterms than full-term infants.

Various reports have suggested that prolonged seizures may lead to poor neurodevelopmental outcome [2–4]; thus appropriate treatment of neonatal seizures is important in reducing long-term neurologic disabilities. Conventionally, the first-line anti-seizure medication for the treatment of neonatal seizures is phenobarbital (PB), despite suboptimal efficacy (only around 50%) and altered pharmacodynamics effects in neonates. Furthermore, currently, a consensus has not been reached regarding the second-line anti-seizure medication such as fosphenytoin, benzodiazepine, levetiracetam (LEV), or lidocaine [5, 6].

LEV is a relatively new and wide spectrum anti-seizure medication which is designed to act through synaptic vesicle glycoprotein 2A (SV2A), which is a protein believed to be involved in the release of neurotransmitters [7]. LEV has a unique mechanism of action, novel structure, and very favorable pharmacokinetic and safety profile in neonates [8, 9]. However, insufficient data are available on the efficacy or safety of LEV in preterm infants with seizures. The purpose of this study was to examine the safety and efficacy of LEV for controlling seizures in preterm infants.

## Methods

We performed a retrospective chart review of neonates treated in the neonatal intensive care unit at Seoul St. Mary’s Hospital from January 2013 to January 2015. Infants were eligible for inclusion if they met the following criteria: 1) preterm birth (< 37 weeks); and 2) received LEV during the neonatal period (0 to 28 days after the birth). Exclusion criteria were: 1) infants whose seizures were caused by electrolyte disturbances (i.e., hypoglycemia, hyponatremia, or hypocalcemia): or 2) infants whose seizures were responsive to pyridoxine. We retrospectively analyzed 37 preterm neonates (18 males and 19 females) who received LEV monotherapy or combination therapy including LEV. Neonatal seizures were diagnosed based on clinical manifestations, neurological and physical examinations, laboratory findings, and continuous video-electroencephalography (EEG) findings during the ictal period for at least 48 h. All patients had electroencephalographic confirmation of seizures and continuous monitoring until seizure control (ranging from 48 h to 8 days).

The onset of seizures was classified as occurring within 24 h, between 24 to 48 h, between 2 and 7 days, and over 7 days to 4 weeks after birth. Types of seizures were defined according to Volpe’s classification, including subtle, tonic, clonic, myoclonic, and mixed types [10].

Anti-seizure medications were considered effective if the seizure terminated within one hour after administration without recurring at least 48 h. Anti-seizure medications were considered safe and tolerable if patients had no neurological/physical abnormalities during treatment period and showed no changes in vital signs, electrocardiography, or clinical laboratory parameters. Laboratory parameters included whole blood cell counts, electrolytes, liver enzyme, gas analysis, and ammonia. LEV was mixed with normal saline and initially loaded intravenously (IV) over one hour. After the initial loading, LEV was administered twice daily. Additional application of PB (IV) was used as the second anti-seizure medication, and phenytoin (IV), midazolam (IV), topiramate (orally), or valproic acid (orally) was used as the third anti-seizure medication.

All patients underwent Bayley scales of infant developments (3rd edition) at 1 year after birth and developmental delay was defined as significant delay (> 2 standard deviation below age-matched peers) in the following areas: gross and fine motor; speech and language; cognition; personal and social development. This study was approved by the institutional review board (KC12RAS10602) of The Catholic University of Korea, Seoul St. Mary’s Hospital, Korea.

## Results

### Gestational ages and body weights

The gestational age of 37 preterm infants ranged from 26 to 36^+ 6^ weeks. Their birth weights ranged from 590 g to 3300 g (Table 1). Three infants (8%) had extremely low birth weights (less than 1000 g), 10 (27%) had very low birth weights (less than 1500 g), 14 (38%) had low birth weights (2499 g or less), and 10 (27%) had normal birth weights (2500–4200 g).

**Table 1:** Demographic features of patients

		Number of patients (*n* = 37, %)
Sex	Male	18 (49)
	Female	19 (51)
Gestational age	Mean	31.5 ± 1.9 weeks
	< 28^+ 0^ weeks	5 (14)
	28 > to 36 ^+ 6^ weeks	32 (86)
Birth weight	Mean	1840 ± 102 g
	< 1000 g	3 (8)
	1000~ 1500 g	10 (27)
	1500~ 2500 g	14 (38)
	> 2500 g	10 (27)

### Onset of neonatal seizures

Neonatal seizures occurred within 30 min to 45 days after birth (according to gestational ages: from 27 to 44 weeks). Three infants (8%) had seizures within 24 h, 7 infants (19%) had seizures within 48 h, 14 infants (38%) had seizures within 7 days, and 13 infants (35%) had seizures after 7 days. Twenty-four infants (65%) among all the patients showed seizures within one week after birth.

### Types of neonatal seizures

Subtle seizures occurred in more than half of the participants (19 infants, 51.4%). Other types of seizures included clonic (5 infants, 17%), tonic (7 infants, 19%), myoclonic (1 infant, 2%), and mixed seizures (5 infants, 13%).

Nine infants (47%) showed pedaling motions or swimming movements of upper extremities, 6 infants (31%) showed apnea and blood pressure elevation, 2 infants (11%) showed ocular movements or nystagmus, and 2 infants (11%) showed chewing motions.

### Brain MRI findings

Brain sonogram or magnetic resonance imaging (MRI) was performed on all preterm infants and 35 infants (94%) showed abnormal findings. The most common finding was hypoxic ischemic encephalopathy (HIE) (15 infants, 41%). Six infants (16%) had germinal matrix hemorrhage, 14 infants (38%) had intraventricular/subdural/subarachnoid hemorrhage, 1 infant had cortical dysplasia (3%), and 1 infant (3%) had bacterial meningitis.

### Response to anti-seizure medication

**Fig. 1:**
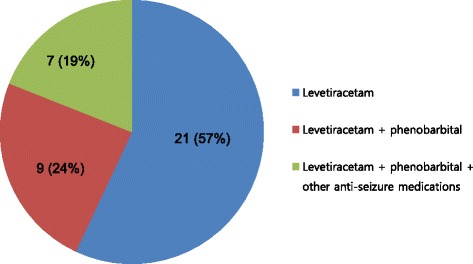
Response of neonatal seizures to anti-seizure medications (*N* = 37)

LEV was administrated intravenously (IV) as the first-line anti-seizure medication and PB was used as the second anti-seizure medication (Fig. 1). LEV (40–60 mg/kg, mean dose 56 mg/kg) was mixed with normal saline and initially loaded over one hour; after initial loading, LEV (20-30 mg/kg, mean dose 23 mg/kg) was administered twice per day. For infants who continued to have seizures after LEV administration, additional infusion of PB (IV, 20 mg/kg) was used as the second anti-seizure medication, and phenytoin (IV, 20 mg/kg), midazolam (IV continuous infusion, 0.1–0.3 mg/kg/h), topiramate (orally, 5–8 mg/kg), or valproic acid (IV, 10–15 mg/kg) was used as the third anti-seizure medications. Overall, seizures terminated in 21 of 37 of the neonates (57%) after receiving LEV, and no additional anti-seizure medications were required (Fig. 1). Sixteen infants (43%) required additional anti-seizure medications during LEV treatment. Seizure cessation was achieved 9 infants (24%) after administration of both LEV and PB. The remaining 7 infants (9%) continued with seizures until a third line anti-seizure medication (phenytoin, topiramate, midazolam, or valproic acid) were administered.

Twenty-one infants (57%) were seizure free under LEV by the end of the first week. Of the remaining, 13 (35%) were seizure free by the 5th week, and 3 (8%) were seizure free by the 11th week after initiating the treatment. In 19 infants (51%), LEV was discontinued after 2–4 weeks of being seizure free.

The mean duration of anti-seizure medication therapy was 42 ± 21 days (range, 15 to 189 days); 36 ± 17 days under LEV monotherapy and 54 ± 21 days under polytherapy. Twenty-three patients (62%) were discharged with LEV, 11 patients (30%) were discharged with additional anti-seizure medications, and 3 patients (8%) were discharged without anti-seizure medication.

### Adverse reactions of LEV

There were no documented adverse drug reactions during or after LEV administration. No serious or consistent emergent laboratory abnormalities were observed. All patients were monitored for increased irritability or somnolence; however no clear adverse reactions including rash and tremor were observed during the study.

### Follow-up and neurological outcomes

Physical, neurological, and developmental examinations were performed for all patients at 1 year after birth. There are no mortality in 37 infants. Twenty-eight patients (75%) were free of seizures and had been completely weaned off all anti-seizure medications. Fourteen infants (38%) showed developmental delay, and 9 infants (25%) had post-neonatal epilepsy with receiving anti-seizure medications.

## Discussion

Neonatal seizures are neurological emergencies and must be treated promptly since seizures can aggravate neuronal injury in the immature brain and contribute to the pathogenesis of later neurodevelopmental disabilities or post-neonatal epilepsy [2]. There are only a few treatment options in neonates with seizures because anti-seizure medications such as PB, phenytoin, and benzodiazepines are rarely effective in controlling epileptogenesis in neonates, and the known risk of adverse effect on cognition effect of PB in infants [11].

Levetiracetam is an anti-seizure medication originally approved for the use in children 4 years old and above in 2005. It received approval for the administration to infants 1 month old (for oral application) and above in 2011. Favorable features of LEV include linear pharmacokinetics, rapid absorption (within 30 min), nonhepatic elimination, lack of protein binding (< 10%), no drug-drug interaction with other medications, and relatively short half-life compared to PB [7]. The availability of its oral and intravenous formulations has made it very useful as an off-label anti-seizure medication in the treatment of neonatal seizures. It has been found that LEV does not increase apoptosis in a developing rodent brain or interfere with neuroprotective up-regulation of hypoxia inducible transcription factor 1 (HIF-1a) [12]. It can also decrease neurodegeneration in rodent models of hypoxic-ischemia [13] or epilepsy [14]. Due to maturational differences and adverse complications of the brain, preterm infants of less than 30 weeks of gestation have a higher incidence of seizures compared to neonates older than 30 weeks [15]. Seizures are a sign of neuronal injury and approximately 40%–60% of all neonatal seizures are attributable to HIE [2]. In our study, the most common brain MRI finding was also HIE (41%). Repeated seizures may be deleterious to the developing brain without disturbing ventilation or perfusion by increasing central nervous system metabolic demand, leading to the release of excitatory amino acid such as glutamate [16]. Neonatal seizures have not been directly shown to lead to neuronal cell death and they are clearly associated with alterations in neurogenesis or synaptic organization [17]. Therefore, continued seizures have a poor prognosis and may lead to serious sequelae such as intellectual disability and motor deficit.

Studies using animal models have shown that LEV does not lead to neuronal apoptosis in immature brain or disrupt synaptic development and may even have neuroprotective properties [18]. Kilicdag et al. [19] demonstrated that administration of LEV after hypoxia significantly reduced the number of apoptotic cells in the cerebral cortex and hippocampus compared to the control in an animal model (*p* < 0.006). When given prophylactically to HIE-induced neonatal rodents, LEV can significantly reduce seizure activity and duration of ictal EEG activity [20]. These findings suggest that LEV may be a viable choice for treating neonatal seizures in HIE. Decreased efficacy and adverse neurodevelopmental prognosis of conventional anti-seizure medications have generated an interest in the use of LEV for the treatment of neonatal seizures.

Despite published data on children and adolescents, only a few have evaluated the efficacy and safety of LEV in neonatal seizures. Several studies have shown the efficacy and safety of LEV in neonatal seizures with diverse etiologies over the past decade [21–32] (Table 2). These studies have demonstrated that LEV may be effective in neonatal seizures when conventional anti-seizure medications have failed. Overall results have shown that LEV may have an overall effect of 50%–80% seizure reduction. These data suggest that LEV can be effective for the treatment of neonatal seizures [25, 26], with a favorable tolerability and side-effect profile comparable to PB. In our study, the use of LEV as the first anti-seizure medication demonstrated seizure cessation within 24 h in 57% (21/37) of patients. Ramantani et al. [24] have also reported the efficacy of LEV in a prospective study using LEV as the first anti-seizure medication. Thirty neonates (79%) including preterm infants were seizure-free under LEV by the end of the 1st week. Such variations in LEV efficacy may be due to the difference in etiology, types of seizures, methods of diagnosis, definitions of efficacy, use of concomitant anti-seizure medications, and dose of LEV.

**Table 2:** Levetiracetam studies in neonatal seizures

Study	Year of publication	No. of patients	Gestational age	Anti-seizure medications used before LEV	LEV dose	Results	Remarks
Shoemaker et al. [21]	2007	3	25^+ 6^–41^+ 6^ weeks	PB, MDZ, fPHT	LD: 60 mg/kg (oral) MD: 30 mg/kg	All 3 patients showed seizure-free with LEV monotherapy.	
Furwentsches et al. [22]	2010	6	37^+ 1^–41^+ 3^ weeks	PB	ID: 10 mg/kg MD: 50 mg/kg	All 6 patients showed seizure-free within 6 days.	Prospective
Ledet et al. [23]	2010	1	40^+ 5^ weeks	PB	LD: 40 mg/kg, MD: 40 mg/kg	Seizure-free	Acute lymphoblastic leukemia
Ramantani et al. [24]	2011	38	28–36^+ 6^ weeks	none	ID: 10–20 mg/kg MD:45–60 mg/kg	Thirty infants (79%) seizure-free under LEV at the end of 1st week.	Prospective
Khan et al. [26]	2011	22	37^+ 5^–41^+ 2^ weeks	PB, fPHT	LD: 10–50 mg/kg MD: 50 mg/kg	Nineteen of 22 patients (86%) demonstrated immediate seizure cessation at 1 h.	LEV as 1st AED in 3 patient
Abend et al. [27]	2011	23	35–41 weeks	PB, PHT	ID: 5–22 mg/kg MD: 10–80 mg/kg	50% seizure reduction in 35% (8/23), including seizure termination in 7 (30%)	LEV as 1st AED in 4 patient
Sharpe et al.	2012	18	37–41 weeks	PB	LD: 20–40 mg/kg MD: 5–10 mg/kg	Six (33%) of the 18 required no additional AEDs after LEV administration.	
Rakshasbhuvankar et al. [28]	2013	8	22^+ 6^-ferm weeks	PB, PTH, MDZ, CZP	ID: 10 mg/kg MD: 10–35 mg/kg	Six (75%) of the eight neonates had an excellent response with either cessation of reduction in seizures by at least 80%.	
Khan et al. [25]	2013	12	23^+ 3^–36 weeks	PB	LD: 25–50 mg/kg MD: 50 mg/kg	Nine of 11 patients (82%) reached seizure cessation within 24 h of receiving LEV.	LEV as 1st AED in 3 patient
Kirmani et al. [29]	2014	22	NA	NA	LD: 10–50 mg/kg MD: NA	Seizure cessation was achieved in 86% (19/22) of patients one hour after the loading dose.	
Neininger et al. [32]	2015	72	23^+ 2^–41^+ 7^ weeks	PB, PTH, MZD, DIZ	ID: 4.9–106.2 mg/kg (mean 20.3 mg/kg) MD: 41.7 mg/kg		LEV as 1st AED in 15 patient,
Shin et al. [30]	2017	18	24^+ 3^–40^+ 2^ weeks	PB, PHT	ID: 4.9–59.5 mg/kg (mean 20 mg/kg) MD: 4.5–99.5 mg/kg (mean 29 mg/kg)	Seventeen patients (94%) had seizure cessation within 1 week and 16 (84%) remained seizure-free at 30 days under LEV therapy.	LEV as 1st AED in 1 patient
Venkatesan et al. [31]	2017	32	35–42^+ 4^ weeks	PB	LD: 20–150 mg/kg (mean 63 mg/kg) MD: 30–100 mg/kg (mean 65 mg/kg)	Thirty two neonates received LEV after PB, the seizures stopped in 27 (84%) patients.	LEV as 1st AED in 2 patient
Our study		37	26–36^+ 6^ weeks	None	LD: 40–60 mg/kg MD: 40–60 mg/kg	Seizures were stopped in 21 (57%) of 37 of neonates after receiving LEV, and no additional AEDs were required	

*PB* phenobarbital, *MDZ* midazolam, *PHT* phenytoin, *fPHT* fosphenytoin, *CZP* clonazepam, *DIZ* diazepam, *LD* loading dose, *MD* maintenance dose, *ID* Initial dose, *NA* not available

Anti-seizure medications such as NMDA receptor antagonists (i.e., ketamine or felbamate), GABA agonists (i.e., PB or benzodiazepine), and sodium channel blockers (i.e., phenytoin or carbamazepine) have been considered to potentiate neurodegeneration in the developing brain of animal models [33]. Dissimilar to other conventional anti-seizure medications, LEV has shown favorable neurodevelopment outcomes and lack of neurodegenerative effects in early animal studies, making LEV a fair treatment option in neonatal seizures. Because SV2a is found in the whole brain, LEV can be used to treat multiple types of seizures that arise from various regions of the brain, as seen in neonatal seizures.

Our study also monitored safety parameters including vital signs and laboratory findings. None of these parameters revealed any adverse effects following LEV administration. These findings have motivated us to institute a protocol for neonatal seizures including preterm infants. Furthermore no serious adverse reactions were seen in our patients. These results were consistent with the most of the data previously reports. A 2007 survey showed that 47% of pediatric neurologists recommended LEV’s off-label use for treating neonatal seizures [34]. Although LEV seems to be safe and effective in neonatal seizures, whether its use will be approved as the first-line anti-seizure medication or as monotherapy remains unclear. Moreover, the pharmacokinetics and safety of LEV appears to be more reasonable than those of conventional anti-seizure medications (i.e., PB, phenytoin or benzodiazepine) in neonatal seizures. Larger prospective studies in newborn patients are needed to investigate LEV as an effective and safe treatment choice for neonatal seizures.

Our results suggest that LEV is a potentially good option to as the first-line anti-seizure medication for treating neonatal seizures including preterm infants. However, our study had some limitations, including a small sample size and its retrospective nature. Our data showed a favorable profile of LEV in neonatal seizures, but additional future researches are needed to clarify its efficacy and safety. Also, long-term observation of neurodevelopmental outcome is necessary in order to establish a proper alternative to traditional anti-seizure medications in neonates with seizures.

## Conclusion

Although this study does not provide conclusive evidence for using LEV in neonatal seizures, treatment with LEV did not result in any adverse reactions and was generally well tolerated. Our data will aid in the decision for using LEV as a treatment option in preterm infants with neonatal seizures, which can improve neurological outcome. Further research studies with larger datasets of prospective randomized control study on LEV use are needed to clarify the efficacy and safety of LEV.

## Abbreviations

EEG: Electroencephalography
HIE: Hypoxic ischemic encephalopathy
LEV: Levetiracetam
MRI: Magnetic resonance image
PB: Phenobarbital
